# Ethnobotanical Study of Medicinal Plants Used to Treat Human and Livestock Ailments in Hulet Eju Enese Woreda, East Gojjam Zone of Amhara Region, Ethiopia

**DOI:** 10.1155/2021/6668541

**Published:** 2021-03-29

**Authors:** Birhanu Adibaru Abebe, Samuel Chane Teferi

**Affiliations:** College of Natural and Computational Sciences, Department of Biology, Salale University, Fiche, Ethiopia

## Abstract

Indigenous people of a given community have their own local specific knowledge on plant use, management, and conservation. The objective of this study was to document medicinal plants used to treat human and livestock ailments in Hulet Eju Enese Woreda. The data were collected using semistructured interviews, focus group discussions, and field observations with local people. A total of 100 informants over the age of 20 years were selected to collect information on medicinal plant use. Descriptive statistics (percentage and frequency), Jaccard's similarity index, independent sample *t*-test and analysis of variance, informant consensus factor, fidelity level, preference ranking, and direct matrix ranking were computed. A total of 80 medicinal plant species belonging to 75 genera and 52 families were documented. In terms of species diversity, Solanaceae stood first with 5 species followed by Euphorbiaceae and Malvaceae with 4 species each. Out of eighty medicinal plants, 53 species were used to treat only human ailments, 8 species were used to treat only livestock ailments, and the remaining 19 species were used for treating both human and livestock ailments. From the total medicinal plant species, shrub constitutes the largest number with 42.5% species. The most frequently used plant parts were leaves, accounting for 28.9%. The major routes of administration were oral accounts, 81 (57%), followed by dermal, 45 (31.7%), nasal, 6 (4.2%), and others, 10 (7%). In the disease category with the highest informant consensus factor (0.83) value was sudden sickness. There was highest preference (49) for *Phytolacca dodecandra* to treat rabies. *Cordia africana* was shown to be the top multipurpose species. This study revealed that the study area was rich in medicinal plants. Agricultural expansion, charcoal making, and firewood collection were considered major threats to medicinal plants. Therefore, awareness creation to the youth and training to the healers play a pivotal role to prevent the loss of indigenous knowledge.

## 1. Introduction

Indigenous people of a given community have their own local specific knowledge on plant use, management, and conservation [1]. This knowledge about plants of their surrounding related to their use, classification, and management practices is generally known as indigenous knowledge or traditional knowledge [2]. One type of such knowledge of indigenous people on plants is related to their use in traditional medicine to treat human and livestock ailments [3]. Even today, plants remain the source of medicine for the majority (80%) of people in developing countries to alleviate health problems, while, in developed countries such as the United States, plant-derived drugs constitute as much as 25% of the total drugs [4]. Moreover, medicinal plants remain the most important and sometimes the only source of therapeutics. Besides their use in preventing and curing various ailments, some medicinal plants are serving as export commodities and source of considerable income for farmers [5].

Medicinal plants are an integral part of the African healthcare system from time immemorial. Africa is blessed with enormous biodiversity resources and it is estimated to contain between 40 and 45,000 species of plants with a potential for development, out of which 5,000 species are used medicinally [6]. Rural Africa is especially bestowed with the most attainable and most reasonably priced phytocosmetics prescribed by traditional healers accessible to the local community and sometimes the only option left for skincare in such remote areas [7]. Many investigators indicate that traditional medicines might offer potential template molecules in the drug discovery process. In vitro and in vivo assays and randomized controlled trials using standardized products or products containing pure plant extracts must be carried out and reported for each claim [8]. For example, findings showed the promising antioxidant, antimicrobial, and antidiabetic activity of the fruits, seeds, and leaves of *Vangueria madagascariensis* [9].

The Ethiopian flora is estimated to contain between 6,000 and 7,000 species of higher plants, of which 12% are endemic, which make the country among the most diverse floristic regions of the world. Ethiopia is also a home to many languages, cultures, and beliefs, which in turn has contributed to the high diversity of traditional knowledge and practices of the people in using medicinal plants [10]. 80% of humans and 90% of livestock in Ethiopia rely on traditional medicine for their primary healthcare systems [4, 11]. Not only Ethiopian population but also around 60% of world population depends on traditional medicine [3]. The reason for high reliance of people in developing countries on traditional medicine is its cultural acceptability, effectiveness against certain type of ailments, accessibility, and affordability as compared to modern medicine [12–14]. Regardless of its contribution to the society, traditional medicine has been given little attention in modern research and less effort has been made to promote the practice in Ethiopia [15]. Due to natural and anthropogenic factors, the biodiversity in general and medicinal plants in particular are being depleted at an alarming rate in the country [16]. The current loss of medicinal plants and associated indigenous knowledge links with environmental degradation, deforestation, agricultural expansion, overexploitation, and population growth are the principal threat to medicinal plants and associated indigenous knowledge in Ethiopia [17]. Loss of indigenous knowledge is also aggravated by the expansion of modern education, making the younger generation underestimate its traditional values. This resulted in the deterioration of traditional practices. As it was reported by [6] in Debre Libanos woreda, Central Ethiopia, knowledge of medicinal plants has been passed orally from one generation to the next by priests and traditional healers.

Identification, documentation, and conservation of medicinal plants and the associated knowledge have been conducted in different parts of Ethiopia. However, study on medicinal plants is not enough when compared to the diverse vegetation and indigenous knowledge the country has. In Amhara region, there is habitat and species loss due to continued deforestation and agricultural expansion, as well as loss of associated indigenous knowledge [18]. There are no previous studies regarding ethnobotanical studies in Hulet Eju Enese woreda. Therefore, this study was proposed to document medicinal plants used to treat human and livestock ailments in Hulet Eju Enese woreda, East Gojjam zone of Amhara region, Ethiopia.

## 2. Materials and Methods

### 2.1. Description of the Study Area

**Figure 1:**
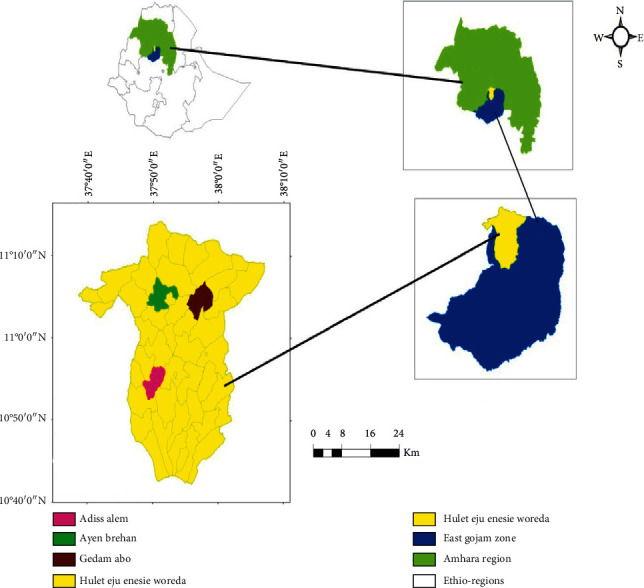
Map of the study site (GIS).

The study was conducted in Hulet Eju Enese district, East Gojjam zone of Amhara regional state, Ethiopia. The district is 120 km east from the capital city of the Amhara regional state, Bahir Dar, and 202 km north from the administrative zone, Debre Markos. It is 363 km to the north of Addis Ababa and it is located between 10° 40′ 00″ to 11°10′ 00″ N latitude and 37°40′ 0″ to 38°10′ 0″ E longitude (Figure 1). The district has an altitude range of 1290–4036 m above sea level The *Sabero Dilde*y (also known as the “Second Portuguese Bridge” or the “Broken Bridge”) crosses the Abay here, connecting Hulet Eju Enese with Este, a woreda in South Gondar [19]. Agroecologically, the district is classified as midland 52% (*“Weinadega”*) (2,387–2555 m above sea level) and has temperature of 18°C–24°C with an average rainfall of 1190 mm per annum, 18% highland (“*Dega*”) (2555–4036 m above sea level) with temperature of 13°C–20°C, and average rainfall of 1260 mm per annum and 30% of the area covered by “*Kola*” (lowland) (1290–2387 m above sea level), which has a temperature of 22°C–28°C with an average rainfall of 1000 mm per annum [19].

The district consists of 40 rural and 6 urban Kebeles and has a total population of 275,638, of which 137,382 are men and 138,256 are women. Out of this number, 30,594 are urban inhabitants with an area of 1,496.69 square kilometers. There are 64,272 households with average of 4.29 persons per household. The majority of the inhabitants practice Ethiopia Orthodox Christianity, with 95.3% reporting that as their religion, while 4.7% of the population are Muslims. The largest ethnic group reported in Hulet Eju Enese is Amhara (99.93%) [20]. The land in the study area is classified into five categories: cultivated (66.7%), grazing (13%), bushes and forest land around homestead (7.2%), and others (12.96%) [19]. The livestock production is one of the major economic bases of the area. The total livestock population in the district is estimated to be 726,157, of which 88,112 (12.12%) are cattle, 488,649 (67.2%) are sheep, 19,579 (2.7%) are goats, 17,183 (2.36%) are equines, and 113,634 (15.62%) are poultry [21].

### 2.2. Reconnaissance Survey and Ethnobotanical Data Collection

A reconnaissance survey was conducted from 1 to 10 August, 2017, to select 3 potential Kebeles. Of the total forty-six Kebeles, Gedam Abo (1700 m above sea level), Ayen Birhan (2,487 m above sea level), and Addis Alem (3410 m above sea level) were selected from lowland, midland, and highland, respectively. Those Kebeles were selected purposively based on the availability of traditional medicine practitioners, traditional medicine use history, and altitudinal variation between the Kebeles. Prior to ethnobotanical data collection on medicinal plants, respondents were selected from the selected Kebeles. Once respondents were selected, ethnobotanical data on medicinal plants were collected from 100 respondents (aged >20), of which 55 (36 men and 19 women) were nonhealers and 45 (34 men and 11 women) were key informants (traditional healers) available for this study (Supp. File Table 4). Key informants were selected based on the information gathered from the local people, while other respondents were randomly selected. Semistructured interviews, group discussions, and guided field walks with key informants for field observations are methods of data collection [22]. First key informants were interviewed individually in order that they mention about the local names of the plants, diseases treated, plant parts used, methods of preparation of remedies, route of application of the remedies, dosage, and factors that threaten medicinal plants. Similarly, the same procedure was followed with randomly selected nonpractitioners of traditional medicine. Based on the checklist prepared, group discussion was made with key informants and field visit was made with them for on-site observation and collection of the plants. The collected voucher specimens were pressed and dried for identification. For some species, preliminary identification was done in the field using illustrations, after which further identification of all specimens was done by comparison with authentic specimens, illustrations, and taxonomic keys from flora of Ethiopia and Eritrea.

### 2.3. Data Analysis

A descriptive statistical method (e.g., percentage and frequency) was employed to summarize ethnobotanical data and statistical test was done using SPSS version 20. Differences in traditional medicinal knowledge due to gender were analyzed using independent *t*-test, but age group and educational level were analyzed using analysis of variance (one-way ANOVA) by using number of medicinal plants reported as a dependent variable and gender, age group, and education level as independent variables.

Jaccard's similarity index was calculated to compare similarity of medicinal plant knowledge between purposively selected Kebeles of different altitude. For this, presence of a given plant species and its utility as medicine or its absence/not considered as medicine are used as datasets [23].

(1) $$\begin{array}{c} JI=\frac{c}{a+b+c}. \end{array}$$

JI is the Jaccard similarity index, “c” is the number of species shared by the study Kebeles, “*a*” is the number of species in Kebelle A only, and “*b*” is the number of species in Kebelle B only. The JI values range between 0 and 1, whereby a value of 1 indicates complete similarity. Informant consensus factors were calculated for categories of ailments to identify the agreements of the informants on the reported cures using the formula used by [24]. ICF was calculated as follows: number of use citations for each ailment minus the number of species used for that ailment, divided by the number of use citations for each ailment minus one.

(2) $$\begin{array}{c} ICF=\frac{n_{\text{ur}}-n_{t}}{n_{\text{ur}}-1}, \end{array}$$

where *n*_ur_ is the number of use citations for each ailment and *n*_t_ is the number of species used for that ailment. The fidelity level (FL), the percentage of informants claiming the use of a certain plant for the same major purpose, was also calculated for the most frequently reported diseases or ailments using the following equation [25]:

(3) $$\begin{array}{c} \text{FL}\left(\%\right)=\frac{\text{NP}}{N}x100, \end{array}$$

where NP is the number of informants that claim the use of a plant species to treat a particular disease, and *N* is the number of informants that use the plants as a medicine to treat any given disease. Preference ranking was conducted following [1, 2]. For this, ten informants were selected to identify the best preferred medicinal plant species for treatment of a specific disease and preference ranking of seven medicinal plants was conducted for treating rabies. Each informant provided with mentioned medicinal plants reported to cure the illness with leaves of medicinal plant used being paper tagged then asked to assign the highest value (7) for the most preferred species against the illness and the lowest value (1) for the least preferred plant and in accordance of their order. The value given to each species was summed up and the rank for each species was determined based on the total score. Direct matrix ranking exercise was done following [1, 2] in order to compare multipurpose use of a given species so as to relate to the probable pressure exerted on that species by the local people. Based on information gathered from informants, multipurpose tree species were selected out of the total medicinal plants and use diversities of these plants were listed for selected key informant to assign use value to each species. Each key informant then was asked to assign use values (5 = best, 4 = very good, 3 = good, 2 = less used, 1 = least used, and 0 = not used). Accordingly, each key informant's use values were summed up and ranked.

## 3. Results and Discussion

### 3.1. Sociodemographic Profile of Informants

The distribution of informants with respect to age class shows that the highest number of informants was obtained in the age group between 41 and 60. 70 of the total informants were male and 30 were females. Regarding educational status, hundred informants were as follows: 54 illiterate, 24 writing and reading, 15 primary school, and 7 secondary school and above (Table 1).

### 3.2. Medicinal Plants of the Study Area

Eighty medicinal plants species were identified for the treatment of human and livestock ailments distributed across 52 families and 75 genera. Compared to the previous studies, the current study reported relatively the same number of medicinal plant 6species. For instance, 91 species [26], 71 species [27], 83 species [25], and 67 species [28] were reported. Out of the collected medicinal plants, 53 species were used to treat only human ailments, 8 species were used to treat only livestock ailments, and the remaining 19 species were used for treating both human and livestock ailments (Supp. File Table 1). This study agrees with the finding in [5], in which more species were used to treat human ailments than livestock ailments. The possible reasons could be attributed to the relative preference to and emphasis of the people on human health problems as compared to livestock health problems. The data collected from the study site showed that 49 medicinal plants were collected from forest site and river side, 17 species of medicinal plants were collected from agricultural and grazing lands, and the remaining 14 species were collected from home gardens/around home (Supp. File Table 2). Similar studies on Ethiopian medicinal plants also showed that traditional medicinal plants are harvested more from wild habitats than from home gardens [13, 15, 29]. This finding also shows that contribution of medicinal plants from home garden appears minimum compared to forest and other habitats and it needs to be emphasized.

In terms of species diversity, Solanaceae stood first with 5 species, which dominated the medicinal plants, followed by Euphorbiaceae and Malvaceae with 4 species each, Cucurbitaceae, Rutaceae, and Verbenaceae each with 3 species, and Acanthaceae, Apocynaceae, Boraginaceae, Celastraceae, Fabaceae, Lamiaceae, Moraceae, Myrsinaceae, Myrtaceae, Oleaceae, and Polygonaceae each with 2 species. The remaining 36 families were represented by one species each (Supp. File Table 1). In contrast to the present finding, other researches in Ethiopia reported Fabaceae as the most dominant medicinal plant family [16, 30–32].

**Figure 2:**
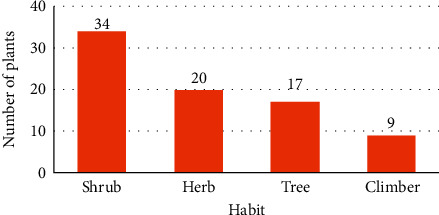
Proportion of the different plant life forms.

Regarding the habit diversity of the reported medicinal plants, majority of them were shrubs followed by herbs, trees, and climbers (Figure 2). The present finding agrees with previous reports [29, 33–36]. The dominance of shrubs as medicinal plants of the study area is because they can be harvested year round compared to herbs, which are short lived, while tree and climbers were rarely found in the study area. However, reports elsewhere showed that herbs were the frequently used medicinal plants, which have a higher relative abundance as compared to other life forms [25, 37, 38].

### 3.3. Plant Parts Used, Preparation, and Route of Administration of Medicinal Plants

**Figure 3:**
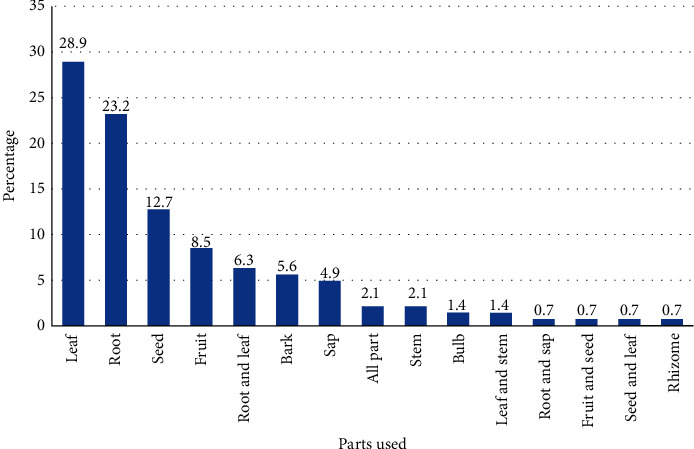
Plant parts used in preparation of remedies.

People of the study area harvest different plant parts for preparation of traditional medicines. However, the mostly used plant part is leaf followed by root, seed, fruit, and so forth (Figure 3), which is an important finding because harvesting leaves does not have detrimental effects on the survival of the medicinal plants, whereas harvesting roots and whole plants has a negative impact on the survival. It agrees with other ethnomedicinal studies in Ethiopia which showed leaves as the most frequently used plant part [13, 27, 39–42] followed by roots [15, 26, 29].

**Figure 4:**
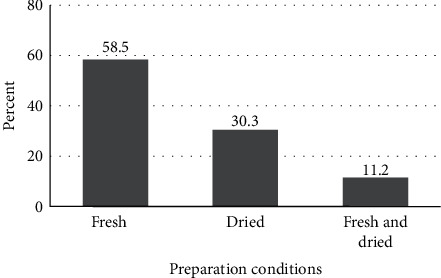
Preparation conditions of herbal remedies.

According to the respondents, herbal remedies are prepared using fresh plant material (58.5%) followed by dried plant material (30.3%) and 11.2% of them reported the use of plant materials in fresh or dried form (Figure 4). Similar finding was reported by [13, 27, 40–43] that the fresh plant material is the most commonly used condition of preparation. Traditional healers claim that some medicinal plants lose their healing potential if not used in fresh condition.

Traditional remedies were prepared in different ways, which actually, according to the respondents, depend on the type and position of the disease. The common ways of remedial preparations reported were crushed, ground, powdered, squeezed, and boiled. The present finding is in agreement with other studies where crushing and squeezing [25, 31] and homogenizing and crushing [37] were the main use forms. This might be due to difference in culture and knowledge across different sociocultural groups. Routes of administration of the remedies also appear to be dependent on part of the body affected and include mainly oral (whereby the patients drink and eat the preparations), followed by dermal (where remedies are creamed, rubbed against the skin, and tied on the skin, or the skin is bathed with the remedies) and nasal (where remedies are sniffed/smoked) routes (Table 2). Ethnomedicinal studies such as those in [28, 40, 43, 44] reported that oral administration is the most commonly used route followed by external/skin creaming.

It was noted that there is no precise dosage for remedies, but people determine the amount in count or volume based on the age and physical condition of the patient. This has been the main drawback of traditional medicine [45]. Hence, there is a need to give priority attention to the establishment of standardized traditional treatment guidelines for medicinal plants by well-known traditional healers. Some of the remedies are taken with different additives and solvents. The additives include butter, honey, milk, *Injera*, sugar, local alcohol (*Tella* or *Arekie*), salt, oil, coffee, and tea. These additives have double function, that is, to get better taste and reduce adverse effects such as vomiting and diarrhea and enhance the efficacy and healing conditions as explained by informants. A similar study was carried out in [46] by Seifu who reported that the Afar people and their traditional healers used solvents and additives like water, honey, sugar, and milk of goat and camels during the preparation of traditional medicines.

### 3.4. Agreement of Respondents on Medicinal Values of Plants and Use Value Ranking

#### 3.4.1. Jaccard's Similarity Index

Analysis of Jaccard's similarity index was conducted using number of medicinal plants reported from each Kebele to show their traditional medicinal plants' knowledge similarity between Kebeles. Jaccard's similarity index (JSI) showed that Ayen Birhan and Gedam Abo have the highest similarity followed by Ayen Birhan and Addis Alem with JSI of 0.91 and 0.77, respectively. The least similarity in study area was observed between Addis Alem and Gedam Abo, which have Jaccard's similarity index of 0.65 (Table 3). This result showed that almost all sites were similar in traditional medicinal plants' knowledge as all of them were related to each other agroecologically and this is also the result of their geographical proximity among the three sites and the same ethnic group with the same cultural background inhabiting in the three Kebeles.

#### 3.4.2. Informant Consensus Factors

All cited human and livestock diseases were categorized into nine categories, namely, blood and circulatory problems, gastrointestinal related disease, malaria, rabies, snake bite, problems of nervous system, problems of respiratory system, problems of urinogenital system, sudden sickness (headache, fever, and fibril illness), sensorial problem (toothache, eye, ear, and nose disease), and skin problem. These diseases were categorized based on the nature of disease, place of attack, and sign and symptoms of diseases. Disease categories with relatively higher ICF values were sudden sickness (0.83), sensorial problem (0.74), blood and circulatory problems (0.67), and skin related problem (Table 4). This indicates the common occurrence of these diseases so that a bigger number of people exchange information and agree on plant species that can be used to treat these diseases compared to the rest (Supp. File Table 3). A high value of ICF (close to one) indicated that the informants rely most on the same taxa to treat a specific disease, while lower value of ICF (close to zero) indicates that the informants disagree to treat a given ailment [47].

#### 3.4.3. Fidelity Level Index

Fidelity level (FL) values were calculated for some commonly used medicinal plants against some commonly reported ailments: *Verbena officinalis* (against stomachache), *Embelia schimperi* (against tapeworm), *Zehneria scabra* (against febrile illness and skin rash*), Rosa abyssinica* (against tapeworm and stomachache), *Datura stramonium* (against dandruff and toothache), *Phytolacca dodecandra* (against gonorrhea, rabies, and anthrax), *Croton macrostachyus* (against febrile illness, ascariasis, and wart), *Kalanchoe laciniata* (against nasal bleeding, body swelling, and leg swelling), and *Justicia schimperiana* (against rabies, gonorrhea, wound, and diarrhea). Fidelity level values in the study area varied from 72% to 100%. Generally, the medicinal plants that are widely used by local people to treat several ailments have less fidelity level value, while medicinal plants used to treat one or few ailments have 100% FL. The result of this study shows that *Verbena officinalis* and *Embelia schimperi* had 100% FL value. These medicinal plants have the highest FL values, which could be an indication of their good healing potential in the study area and the others are below 100%. For example, *Justicia schimperiana* is a widely used species to treat many ailments and its FL is 72% (Table 5). As reported by [26], validation of bioactivity of medicinal plants preferred by traditional healers increases their acceptance both nationally and internationally for healthcare systems. Moreover, the findings of [15, 27] summarized that priority for further pharmacological studies must be given to medicinal plants scoring the highest fidelity level.

#### 3.4.4. Preference Ranking

Preference ranking of seven medicinal plants that were reported for treating rabies was conducted after selecting ten key informants. The informants were asked to compare the given medicinal plants based on their effectiveness and to give the highest number (7) for the medicinal plant which they thought was most effective and the lowest number (1) for the least effective plant in treating rabies. Result showed that *Phytolacca dodecandra* ranked first to treat rabies. Therefore, it is the most effective medicinal plant to treat rabies followed by *Cucumis ficifolius, Argemone mexicana, Euphorbia tirucalli*, and *Rumex nervosus* (Table 6).

#### 3.4.5. Direct Matrix Ranking

Many medicinal plants were reported to have been used in other areas other than medicine. These were used for charcoal making, construction, firewood, fencing, forage, and furniture. Six commonly reported multipurpose species and seven use categories were involved in direct matrix ranking exercise in order to evaluate their relative importance to the local people and the extent of the existing threats related to their use values. The direct matrix ranking result showed that *Cordia africana* ranked first as the most multipurpose medicinal plant by local people for various uses followed by *Eucalyptus globulus, Dodonaea angustifolia, Acacia abyssinica, Croton macrostachyus*, and *Euphorbia abyssinica* (Table 7)*. Cordia africana* is the most threatened species as the informants reported, which is evidently shown by its distribution scarcity and the time required for its collection. Even though *Eucalyptus globulus* is required for various use values and is ranked 2^nd^, it is abundantly recorded in the area. Similarly, the use values reported across the selected species were summed up and ranked. The results show that the local people harvest six multipurpose species mainly for firewood, fencing, medicine, charcoal, furniture, construction, and forage with the following ranking: 1^st^, 2^nd^, 3^rd^, 4^th^, 5^th^, and 6^th^, respectively (Table 7). Medicinal plants with relatively highest use values are considered to be the most used ones and under pressure due to overusage, which may in the long run lead to the rarity of the species. Therefore, high ranking medicinal plants based on their use diversity values need priority attention for conservation [27].

### 3.5. Threats to Medicinal Plants and Associated Indigenous Knowledge

The cause of threats to medicinal plants can be generally grouped into natural and human induced factors. However, as respondents reported in this study, most of the causes for the threats to medicinal plants and the associated indigenous knowledge are the anthropogenic factors such as agricultural expansion, firewood, charcoal, timber, and construction materials. Other researches on threats to medicinal plants in Dale [48], Benna Tsemay district [5], Mana Angetu district [29], Amaro woreda [49], and Wonago woreda [35] indicated findings similar to those of our result. Informants ranked agricultural expansion as the most serious threat to the medicinal plants followed by firewood/charcoal collection and overgrazing (Table 8). As some of the medicinal plants are of multipurpose, their extraction for medicine has also increased the threat. Secrecy in disclosing of the knowledge by traditional healers and negligence of the young generation to learn indigenous knowledge were also noticed as factors that contribute to the loss of indigenous knowledge. Similarly, other findings [15, 27, 50–52] reported that there is an aura of top secrecy in the passing of indigenous knowledge within families. Therefore, creating better conservational awareness to the community members is necessary.

### 3.6. Sociodemographic Factors Influencing Medicinal Plant Knowledge

Statistical analysis showed that gender, age, and educational statuses of respondents were found to influence knowledge of medicinal plants of the local community. Respondents were categorized into 3 age categories; 20–40, 41–60, and above 61 years of age. Analysis of variance (one-way ANOVA) revealed that there was significant (*P*=0.015) difference between age categories in their traditional medicinal plant knowledge. Respondents aged above 61 years reported 5.00 ± 0.49 medicinal plants and those aged between 41 and 60 years reported 4.00 ± 0.29, while those aged below 40 years reported about 3.35 ± 0.23 plants on average. The results of this study show that number of medicinal plants reported by respondents increases with age, and the elders have more accumulated indigenous knowledge on medicinal plants compared to the young generation. This study agrees with [15, 53] in that older people cited more medicinal plant species than younger people. This is the fact that the elders have long experience to use local medicinal plants against different diseases traditionally. But young generations are under the influence of modernization and globalization and were disinterested towards traditional practice. This discovery agrees with the research carried out in Dire Dawa city, eastern Ethiopia [54].

On traditional medicinal knowledge, gender also had significant impact (*P*=0.005, independent *t*-test) where males reported 4.29 ± 0.25 plants and females reported 3.03 ± 0.19 plants. Thus, the present finding revealed that both men and women are knowledgeable on use of traditional plant remedies, in spite of the relative dominance of medicinal plant tradition by men which could relate to the traditional flow of information along the men in the country [55] and elsewhere [56, 57]. Related to educational status, respondents were grouped into four educational levels: illiterate, read and write, primary school, and high school and above. Like age and gender, educational level also had significant difference (*P* < 0.001, ANOVA) in traditional medicinal knowledge. Respondents who can read and write reported 6.67 ± 0.57 medicinal plants, followed by illiterate, 3.46 ± 0.19 plant species, primary school, 2.07 ± 0.20 plants, and 1.86 ± 0.26 medicinal plants, high school and above on average.

## 4. Conclusion and Recommendations

An ethnobotanical study of medicinal plants used by local people of Hulet Eju Enese woreda, East Gojjam zone of Amhara Region, Ethiopia, was conducted. Eighty medicinal plants distributed in 75 genera and 52 families that are used to treat various human and livestock ailments were documented from the study area. From total medicinal plants, the majority were observed in the forest and river side, followed by in agriculture and grazing land and home garden. Out of 80 medicinal plant species, 53 were used to treat only human ailments, 8 species were used to treat only livestock ailments, and the remaining species were used for treating both human and livestock ailments. Analysis of growth forms of these medicinal plants revealed that shrubs are the dominant growth form followed by herb, tree, and climber. Remedies are prepared using fresh plant material. Biggest number of plant parts used for the preparations of remedies were harvested from leaves followed by roots. Most of the medicinal plants are administered orally. The common ways of preparations of traditional medicines were crushed and grounded. Gender, age, and educational status of respondents were found to influence knowledge of medicinal plants of the local community. Therefore, training and awareness about conservation methods should be given to traditional healers and the local community. Moreover, traditional healers should be encouraged to transfer their indigenous knowledge to their family and relatives so as to preserve for the next generation.

## Figures and Tables

**Table 1:** Summary of information about the informants in the study area.

Sex		Age				Educational status		
Male	Female	20–40	41–60	>60	Illiterate	Read and write	Primary school	Secondary school and above
70	30	31	50	19	54	24	15	7

**Table 2:** Route of administration of traditional medicines in the study area.

Route of application	Frequency	Percent (%)
Oral	81	57
Dermal	45	31.7
Nasal	6	4.2
Other	10	7.1
Total	142	100

**Table 3:** Jaccard's similarity index of medicinal plants' knowledge among selected sites.

Kebeles	Ayen Birhan	Gedam Abo	Addis Alem
Ayen Birhan	1	0.91	0.77
Gedam Abo	0.91	1	0.65
Addis Alem	0.77	0.65	1

**Table 4:** Informant consensus factor for major categories of human and livestock diseases.

No.	Categories of ailments	No. of use citations (*n*_ur_)	No. of species (*n*_*t*_)	ICF
1	Sudden sickness (headache, fever, fibril illness, etc.)	53	9	0.83
2	Sensorial problems (toothache, eye, ear, nose disease, etc.)	39	11	0.74
3	Skin problems	69	21	0.71
4	Blood and circulatory problems	28	10	0.67
5	Malaria, rabies, snake bite, etc.	49	20	0.60
6	Problems of respiratory system	28	12	0.59
7	Problems of the urinary system	33	15	0.56
8	Gastrointestinal related disease	47	22	0.54
9	Problems of nervous system	14	8	0.46

**Table 5:** Fidelity level index of the relative healing potential of some selected medicinal plants used against human or livestock ailments.

Botanical name of medicinal plants	Ailment to be treated	NP	N	FL	FL%
*Verbena officinalis*	Stomachache	40	40	1	100
*Embelia schimperi*	Tapeworm	34	34	1	100
*Zehneria scabra*	Fibril illness, skin rash	31	33	0.94	94
*Rosa abyssinica*	Tapeworm, stomachache	26	28	0.93	93
*Datura stramonium*	Dandruff, toothache	20	22	0.91	91
*Phytolacca dodecandra*	Gonorrhea, rabies, anthrax	38	44	0.86	84
*Croton macrostachyus*	Febrile illness, ascariasis, wart	22	26	0.85	85
*Kalanchoe laciniata*	Nasal bleeding, body swelling, leg swelling	12	16	0.75	75
*Justicia schimperiana*	Rabies, gonorrhea, wound	18	25	0.72	72

**Table 6:** Preference ranking of selected medicinal plants based on the degree of their curative power against rabies as perceived by informants.

Respondents	Species						
	*Acanthus sennii*	*Argemone Mexicana*	*Cucumis ficifolius*	*Euphorbia tirucalli*	*Justicia schimperiana*	*Phytolacca dodecandra*	*Rumex nervosus*
*R*_1_	4	6	5	5	1	3	4
*R*_2_	5	4	5	1	3	6	1
*R*_3_	3	5	6	7	2	5	4
*R*_4_	2	7	2	3	3	3	6
*R*_5_	7	3	6	2	2	7	4
*R*_6_	4	4	7	5	5	5	3
*R*_7_	2	7	1	6	6	4	2
*R*_8_	4	4	5	5	4	5	5
*R*_9_	3	3	5	2	5	6	6
*R*_10_	5	1	5	4	2	5	4
Total	36	44	47	40	33	49	39
Rank	6^th^	3^rd^	2^nd^	4^th^	7^th^	1^st^	5^th^

**Table 7:** Direct matrix ranking for six selected plant species and main use in study area.

Plant species	Use categories								
	Medicine	Forage	Fire wood	Charcoal	Fence	Construction	Furniture	Total	Rank
*Croton macrostachyus*	5	2	5	2	4	0	1	19	5^th^
*Eucalyptus globulus*	3	0	5	3	5	5	5	25	2^nd^
*Cordia africana*	2	5	5	3	4	3	5	27	1^st^
*Acacia abyssinica*	3	2	5	5	3	2	0	21	4^th^
*Dodonaea angustifolia*	3	4	5	2	5	3	0	22	3^rd^
*Euphorbia abyssinica*	5	0	4	3	4	0	3	16	6^th^
Total	21	13	29	18	25	13	14		
Rank	3^rd^	6^th^	1^st^	4^th^	2^nd^	6^th^	5^th^		

**Table 8:** Ranking factors threatening medicinal plant species in the study area.

Threat factors	Respondents										Total	Rank
	R1	R2	R3	R4	R5	R6	R7	R8	R9	R10		
Agricultural expansion	4	4	5	5	4	5	5	5	4	4	45	1^st^
Firewood and charcoal	4	5	5	4	4	4	5	4	4	5	44	2^nd^
Overgrazing	5	4	5	5	4	5	4	4	5	4	40	3^rd^
Drought	4	4	3	4	5	4	4	3	4	3	38	4^th^
For construction	3	4	3	4	3	3	4	4	3	5	36	5^th^
For medicinal value	1	2	3	2	1	2	3	2	3	3	22	6^th^

Ranking was done based on the use criteria rated as 5 = extremely high, 4 = very high, 3 = high, 2 = low, and 1 = very low.

## Data Availability

The dataset used to support the findings of this study is included within the text and supplementary materials.

## Supplementary Materials

Supplementary Materials Supplementary Table 1: list of medicinal plants used to treat human and livestock ailments in the study area [with scientific name, family name, local name, habit, used for, parts used, disease treated, method of preparations, and route of application]. Note: all local names are in Amharic language and the number in parenthesis with common name is collection number. Supplementary Table 2: habitat of medicinal plant species in the study area. Supplementary Table 3: health problems treated by traditional medicinal plants in the study area. Supplementary Table 4: total number of informants in the study area in each Kebele.
