# Supplementary material for: Ethnobotanical Study of Medicinal Plants Used to Treat Human and Livestock Ailments in Hulet Eju Enese Woreda, East Gojjam Zone of Amhara Region, Ethiopia
Source: Evid Based Complement Alternat Med. 2021 Mar 29;2021:6668541. doi: 10.1155/2021/6668541 (PMC8021471; doi:10.1155/2021/6668541)
Supplement: Supplementary Materials — Supplementary Table 1: list of medicinal plants used to treat human and livestock ailments in the study area [with scientific name, family name, local name, habit, used for, parts used, disease treated, method of preparations, and route of application]. Note: all local names are in Amharic language and the number in parenthesis with common name is collection number. Supplementary Table 2: habitat of medicinal plant species in the study area. Supplementary Table 3: health problems treated by traditional medicinal plants in the study area. Supplementary Table 4: total number of informants in the study area in each Kebele. [file 6668541.f1.docx]

**Raw Data (Supplementary file)**

**Table 1: List of Medicinal Plants Used to Treat Human and Livestock Ailments in the Study Area; [**with scientific name, family name, local name, habit, used for, parts used, disease treated, and method of preparations and route of application]. **Note:** All local names are in **Amharic** language and the number in parenthesis with common name is collection number.

**Key;** Hu= Human, Li =live stocks, HL= human and live stocks, Ay=Ayenbirhan, Ge=Gedamabo, Ad=Addisalem, D=dry, F=fresh.

| **No** | **Botanical name and plant habit** | **Family name** | | **Local name**  **and collection No** | | | **Used for** | | | | **Ailments** | | **Part used and mode of preparation of the remedies** | **Route of application** |
| --- | --- | --- | --- | --- | --- | --- | --- | --- | --- | --- | --- | --- | --- | --- |
| 1 | *Acacia abyssinica* Hochst. Ex Benth . **Tree** | Fabaceae | | Girar  (Ad71) | | | Hu | | |  | Stomachache | | **Root F:** The fresh root of *Acacia abyssinica* is powdered; mix withSour milk or “ergo” and drunken one cup before food until treated. | Oral |
|  |  |  |  |  |  |  | Hu | | | | Insect bite | | **Bark F:** The insider part of the fresh bark of *Acacia abyssinica* is ground and tied on the bitted body part. | Dermal |
| 2 | *Acanthus sennii* Chiov. **Shrub** | Acanthaceae | | Kusheshile  (Ay5) | | | Li | | | | Rabies | | **Root D:** The root of *Acanthus sennii* with the root of *Rumex nervosus* and *Grewia ferruginea* grounded together and mix with milk then given to dog. | Oral |
| 3 | *Achyranthes aspera* L. **Herb** | Amaranthaceae | | Telenj  (Ay69) | | | Hu | | | | Wound  (Liffe) | | **Root F:** Crushed and powdered, mixed with honey & tied for three days then with butter same day. | Dermal |
|  |  |  | |  | | | Li | | | | Bleeding | | **Above ground F:** Above ground part | Dermal |
|  |  |  | | *Table1: Continued* | | | | | | |  | |  |  |
| **No** | **Botanical name and plant habit** | **Family name** | | **Local name**  **and collection No** | | | **Used for** | | | | **Ailments** | | **Part used and mode of preparation of the remedies** | **Route of application** |
|  |  |  | |  | | |  | | | |  | | of *Achyranthes aspera* is Crushed and tied on bleeding part of all animals. |  |
| 4 | *Acokanthera schimperi* (A.DC.) Schweinf. **Tree** | Apocynaceae | | Merz  (Ge28) | | | Hu | | | | Liver Problem | | **Leaf F:** The leaf of *Acokanthera schimperi* is crushed, powdered, mixed with honey and then eaten about three-four days before food. | Oral |
| 5 | *Allium sativium L* **Herb** | Liliaceae | | Nechshinkurt (Ge26) | | | Hu | | | | Malaria | | **Bulb F:** The bulb of *Allium sativum* with seed of *Lepidium sativum* is powdered together and mixed with little water then drunk or eaten by Injera. | Oral |
|  |  |  |  |  |  |  | Hu | | | | Common cold | | **Bulb F:** The bulb of *Allium sativium* crushed and capture by nose or eaten by Injera. | Nasal/Oral |
| 6 | *Aloe macrocarpa* Tod, **shrub** | Aloaceae | | Eret  (Ay75) | | | Hu | | | | Snake bite | | **Sap F:** The sap of *Aloe macrocarpa* drunk. | Oral |
|  |  |  |  |  |  |  | Hu | | | | Impotency | | **Root and sap DF:** The root of *Aloe macrocarpa* is crushed powdered by its sap and mixed with butter and tied the whole part of penis. | Dermal |
|  |  |  | | *Table1: Continued* | | | | | | |  | |  |  |
| **No** | **Botanical name**  **and plant habit** | **Family name** | | **Local name**  **and collection No** | | | **Used for** | | | | **Ailments** | **Part used and mode of preparation of the remedies** | | **Route of application** |
| 7 | *Arundo donax L,***Shrub** | Poaceae | | Shenbeko  (Ge37) | | | Hu | | | | Abortion | **Root F:** The root of *Arundo donax* and *Solanum marginatum* seed powder mixed by water and drunk it with a cup of tea for seven days. | | Oral |
| 8 | *Argemone mexicana* L. **Herb** | Papaveraceae | | Yahya eshoh  (Ge21) | | | Li | | | | Rabies | **Root D:** The dried root of *Asparagus africanus* is crushed and given with water to all animals. | | Oral |
| 9 | *Asparagus africanus* Lam, **Climber** | Asparagaceae | | Yeset kest  (Ay10) | | | Hu | | | | Gonorrhea | **Root D:** The roots of *Asparagus africanus* is, grounded and mix with honey and stay for a week then eat it in the morning before food for seven days. | | Oral |
|  |  |  |  |  |  |  | Hu | | | | Toothache | **Root F:** The fresh root of *Asparagus africanus* is chewed during ache time. | | Oral |
| 10 | *Bersama abyssinica* Fresen. **Shrub** | Milianthaceae | | Azamira  (Ay48) | | | Hu | | | | Ascariasis | **Leaf F:** The fresh leaves of *Bersama abyssinica* are squeezed and mix with ground seed of *Cucurbita pepo,* then fused with honey and swallow the mixture. | | Oral |
|  |  |  |  |  |  |  | Hu | | | | Eye disease | **Bark D:** The bark of *Bersama abyssinica* is grounded and mix with butter, then applies on the infected eye. | | Eye |
|  |  | | *Table1: Continued* | | | | | | |  |  | |  |  |
| **No** | **Botanical name**  **and plant habit** | **Family name** | | **Local name**  **and collection No** | | | **Used for** | | | | **Ailments** | **Part used and mode of preparation of the remedies** | | **Route of application** |
| 11 | *Buddleja polystachya* Fresen, **Shrub** | Scrophulariaceae | | Anfar  (Ad8) | | | Hu | | | | Tonsillitis | **Shoot F:** The fresh shoot of *Buddleja polystachya* with shoot of *Rumex nervosus* are crushed and mix together with water then put on center of head. | | Dermal |
|  |  |  |  |  |  |  | HL | | | | Hemorrhoid | **Root and shoot F:** The root and shoot of *Buddleja polystachya* are crushed and grounded with bean, seed of *Lepidium satvum* by water*,* then applied on the affected part. | | Dermal |
| 12 | *Capsicum annuum* L, **Herb** | Solanaceae | | Karia  (Ad41) | | | Hu | | | | Malaria | **Fruit/seed FD:** The fruit/seed of *Capsicum annuum* is pounded, powdered and mixed with little water and drunk or eaten by Injera. | | Oral |
| 13 | *Carissa spinarum* L, **Shrub** | Apocynaceae | | Agam  (Ge55) | | | Hu | | | | Snake bite | **Leaf F:** Fresh leaf of *Carissa spinarum* will be crushed and chewed and 2-3 drops of the sap is taken orally. | | Oral |
|  |  |  |  |  |  |  | HL | | | | Wound | **Root and leaf F:** The root and leaf of *Carissa spinarum* are crushed and grounded with the seed of *Lepidium sativum* by water, then applied on the affected part. | | Dermal |
| 14 | *Catha edulis* (Vahl) Forssk. ex Endl,**Shrub** | Celastraceae | | Chat  (Ay40) | | | Hu | | | | Asthma | **Leaf F:** The leaves of *Catha edulis* are crushed and mix with leaves of coffee then boiled together, after that drunk with honey | | Oral |
|  |  |  | | *Table1: Continued* | | | | | | |  |  | |  |
| **No** | **Botanical name**  **and plant habit** | **Family name** | | **Local name**  **and collection No** | | | **Used for** | | | | **Ailments** | **Part used and mode of preparation of the remedies** | | **Route of application** |
|  |  |  | |  | | | Hu | | | | Snake bite | **Shoot F:** The shoot of *Catha edulis* chewed during bite. | | Oral |
| 15 | *Citrus* limon Burn. f. **Tree** | Rutaceae | | Lomy  (Ay14) | | | Hu | | | | Stomachache | The fruit juice *Citru limons* drunk. | | Oral |
|  |  |  |  |  |  |  | Hu | | | | Athletes foot | Fruit F: The fruit of *Citrus limon* is squeezed and creamed on foot for continuous days. | | Dermal |
| 16 | *Clausena anisata* Willd. Benth.**Shrub** | Rutaceae | | Lmich  (Ay62) | | | HL | | | | Skin rash | **Leaf F:** The leafs of *Clausena anisata*, with leaf of *Grewia ferruginea* will be ground together with water and washed the affected skin. | | Dermal |
| 17 | *Clematis simensis* Fresen, **Climber** | Ranunculaceae | | Azoareg  (Ay54) | | | Hu | | | | Wound/Granule | **Root and leaf D:** The root and leaf of *Clematis simensis* is pounded, powdered and mixed with butter and creamed on the affected part until recovery. | | Dermal |
| 18 | *Clerodendrum myricoides* Hochst*,***Shrub** | Verbenaceae | | Msirch  (Ay2) | | | Hu | | | | Wart | **Leaf F:** The fresh leaves of *Clerodendrum myricoides* are crushed; grounded with seed of *Lepidium sativum,* then mix with salt and apply on the affected part. | | Dermal |
| 19 | *Coffea arabica* L.**Shrub** | Rubiaceae | | Buna  (Ay12) | | | Hu | | | | Diarrhea | **Seed D:** The dried seed of *Coffea arabica* is roasted, boiled and then filtrate one cup and mixed with few drop of oil then drunk. | | Oral |
|  |  | | *Table1: Continued* | | | | | | |  |  | |  |  |
| **No** | **Botanical name**  **and plant habit** | **Family name** | | **Local name**  **and Collection No** | | | **Used for** | | | | **Ailments** | **Part used and mode of preparation of the remedies** | | **Route of application** |
|  |  |  | |  | | | Hu | | | | Fire burn | **Seed D:** The seed of *Coffea arabica* is roasted, crushed, powdered then mix with salt and tied on wound. | | Dermal |
| 20 | *Cordia africana* Lam. **Tree** | Boraginaceae | | Wanza  (Ay1) | | | Li | | | | Eye- problem | Leaf D: The leaves of *Cordia Africana* are burned and the burnt leaves’s ash is insertedinto cattle’s eye with butter. | | Eye |
|  |  |  |  |  |  |  | Hu | | | | Wound/likfit | **Leaf D:** Leaf of *Cordia africana* burned and its ash mixed with butter then creamed on the affected part. | | Dermal |
| 21 | *Croton macrostachyus* Hochst. **Tree** | Euphorbiaceae | | Bisana  (Ge30) | | | Hu | | | | Febrile  Illness | **Leaf F:** Leaf of *Croton macrostachyus* and *Rosa abyssinica* are boiled and fumigated. | | Oral &  Nasal |
|  |  |  |  |  |  |  | Hu | | | | Ascariasis | **Root D:** Root of *Croton macrostachyus* is crushed and grounded, then mix with honey and swallow. | | Oral |
|  |  |  |  |  |  |  | Hu | | | | Wart (chirt) | **Sap F:** The twig or petiole of *Croton macrostachyus* is cut and the sap is smear on the patient of the body. | | Dermal |
| 22 | *Cucumis ficifolius* A. Rich. **Climber** | Cucurbitaceae | | Yemdir enbuay  (Ad50) | | | Li | | | | Rabies | **Root D:** The dried root of *Cucumis ficifolius* and *Solanum incanum* crushed, grounded and mix with milk, then given to animals*.* | | Oral |
|  |  | | *Table1: Continued* | | | | | | | |  | |  |  |
| **No** | **Botanical name and plant habit** | **Family name** | | **Local name**  **and collection No** | | | **Used for** | | | | **Ailments** | **Part used and mode of preparation of the remedies** | | **Route of application** |
|  |  |  | |  | | | Hu | | | | Stomachache | **Root F:** The root of *Cucumis ficifolius* is chewed with salt and swallowed. | | Oral |
|  |  |  |  |  |  |  | Hu | | | | Hemorrhoid | **Root F:** The fresh root of *Cucumis ficifolius* will be pounded and washed on the affected part of the body. | | Dermal |
| 23 | *Cucurbita pepo* L. **Climber** | Cucurbitaceae | | Duba  (Ay17) | | | Li | | | | Expel placenta | **Fruit D:** The fruit *of Cucurbita pepo* chopped and the flesh part is boiled with water and given to cattle, goat and sheep for drinking. | | Oral |
|  |  |  |  |  |  |  | HL | | | | Tapeworm | **Seed D:** Seeds of *Cucurbita pepo* are dried roasted and mix with honey then eaten before food (empty stomach) and stay for six hour without eat food for human, given the dried seed for animals with salt in the morning. | | Oral |
| 24 | *Datura stramonium* L. **Herb** | Solanaceae | | Astenagr (Ay7) | | | Hu | | | | Dandruff | **Leaf F:** Fresh leaves pounded and mix with butter then creamed the affected part/head | | Dermal |
|  |  |  |  |  |  |  | Hu | | | | Toothache | **Seed D:** The fresh seeds of *Datura stramonium* are roasted, boil with milk and fumigated the vapor during bed time. | | Oral |
|  |  |  | | *Table1: Continued* | | | | | | |  |  | |  |
| **No** | **Botanical name and plant habit** | **Family name** | | **Local name**  **and collection No** | | | **Used for** | | | | **Ailments** | **Part used and mode of preparation of the remedies** | | **Route of application** |
| 25 | *Dodonaea angustifolia* L.f.  **Shrub** | Sapindaceae | | Kitkita  (Ad52) | | | Hu | | | | Eczema | **Leaf D:** The leaf of *Dodonaea angustifolia* is burn, pounded, powdered and mixed with  butter and creamed the affected part. | | Dermal |
|  |  |  |  |  |  |  | HL | | | | Bone fracture | **Leaf F:** The fresh leaves of *Dodonaea angustifolia* are tied on fracture bone with frame. | | Dermal |
| 26 | *Dombeya torrida* Lam*.***Shrub** | Sterculiaceae | | Wulkefa  (Ad42) | | | Hu | | | | Toothache | **Root & leaf FD:** The root and leaves of *Dombeya torrid* crushed, pound together and mix with salt then apply on teeth. | | Oral |
| 27 | *Dovyalis abyssinica* A. Rich. **Shrub** | Salicaceae | | Koshim  (Ad46) | | | Hu | | | | Abdominal pain | **Fruit F:** Six to ten fruit of *Dovyalis abyssinica* are eaten before food and does not use milk for a weak. | | Oral |
|  |  |  |  |  |  |  | HL | | | | Intestinal parasites | **Fruit F:** The fruit of *Dovyalis abyssinica* are eaten before breakfast every morning. | | Oral |
| 28 | *Embelia schimperi* Vatke. **Climber** | Myrsinaceae | | Enkoko  (Ad80) | | | Hu | | | | Tapeworm | **Seed DF:** The fruits of *Embelia schimperi* are dried grounded and drink it with tella or eat the freshly seed before food in the morning and stay for five to seven hour. | | Oral |
| 29 | *Eucalyptus globulus* Labill. **Tree** | Myrtaceae | | Bule bahirzaf  (Ay63) | | | Hu | | | | Fibril illness | **Leaf F:** The leaf and young branches are boiled in water and the steam will be inhaled during bed times. | | Oral/Nasal |
|  |  | | *Table1: Continued* | | | | | | |  |  | |  |  |
| **No** | **Botanical name and plant habit** | **Family name** | | **Local name**  **and collection No** | | | | **Used for** | | | **Ailments** | **Part used and mode of preparation of the remedies** | | **Route of application** |
| 30 | *Euclea racemosa*  Hiern. **Shrub** | Ebenaceae | | Dedeho  (Ge39) | | | | Hu | | | Toothache | **Steam F:** The fresh stem of *Euclea racemosa* is chewed. | | Oral |
|  |  |  |  |  |  |  |  | HL | | | Tapeworm | **Leaf D:** The dried leaves of *Euclea racemosa* are crushed, grounded, soaked with water then the filtrate is drunk and given to animals. | | Oral |
| 31 | *Euphorbia abyssinica* Gmel. **Tree** | Euphorbiaceae | | Qulkual  (Ge34) | | | | Hu | | | Jaundice | **Root F:** The root of *Euphorbia abyssinica* crushed ground and immersed in water then drunk or baked with bread and eaten it before food in the morning. | | Oral |
|  |  |  |  |  |  |  |  | HL | | | Rabies | **Sap F:**The latex of *Euphorbia abyssinica* is mixed with milk and given to dog or the latex is backed and give to it. | | Oral |
|  |  |  |  |  |  |  |  | Hu | | | Hemorrhoid | **Sap F:** The latex of *Euphorbia abyssinica* is applied on the affected part. | | Dermal |
|  |  |  |  |  |  |  |  | Hu | | | Wound  (kunchir) | **Sap F:** The milky fluid *Euphorbia abyssinica* is applied on the wound. | | Dermal |
| 32 | *Euphorbia tirucalli* L*.* **Shrub** | Euphorbiaceae | | Qinchib  (Ad74) | | | | Hu | | | Wound  (kunchir) | **Sap F:** The milky fluid *Euphorbia tirucalli* is applied on the wound. | | Dermal |
|  |  | | *Table1: Continued* | | | | | | |  |  | |  |  |
| **No** | **Botanical name and plant habit** | **Family name** | | **Local name**  **and collection No** | | | | **Used for** | | | **Ailments** | **Part used and mode of preparation of the remedies** | | **Route of application** |
|  |  |  | |  | | | | HL | | | Rabies | **Sap F:**The latex of *Euphorbia tirucalli* is mixed with milk and given to dog or the latex is backed with bread and eats it then drinks only local alcohol for two weak. | | Oral |
|  |  |  |  |  |  |  |  | Hu | | | Skin rash | **Sap F:** Sap from *Euphorbia tirucalli* creamed on affected skin. | | Dermal |
| 33 | *Ficus sur* Forssk. **Tree** | Moraceae | | Sholla  (Ay16) | | | | Hu | | | Gonorrhea | **Sap F:** The sap of *Ficus sur* together with that of root *Phytolacca dodecandra, Ficus vasta Dodonaea angustifolia,* and *Rhamnus prinoides* will be pounded and taken with tea or local alcoholic drink. | | Oral |
| 34 | *Ficus vasta* Forssk **Tree**. | Moraceae | | Warca  (HE79) | | | | Hu | | | Fibril illness | **Leaf F:** The fresh leaves of *Ficus vasta* is boiled and fumigated during bed time. | | All body |
| 35 | *Foeniculum vulgare* Miller. **Herb** | Apiaceae | | Ensilal  (Ay10) | | | | Hu | | | Cough | **Leaf F:** The fresh leaves of *Foeniculum vulgare* is boiled with tea and drink. | | Oral |
|  |  |  |  |  |  |  |  | Hu | | | Asthma | **Leaf and stem F:** Leaf and stem are crushed,boiled together with milk and drunk. | | Oral |
|  |  |  |  |  |  |  |  | Hu | | | Urinary Retention | **Root F:** The root of *Foeniculum vulgare* is chewed and swallowed the solution. | | Oral |
|  |  | | *Table1: Continued* | | | | | | |  |  | |  |  |
| **No** | **Botanical name and plant habit** | **Family name** | | **Local name**  **and collection No** | | | | **Used for** | | | **Ailments** | **Part used and mode of preparation of the remedies** | | **Route of application** |
| 36 | *Grewia ferruginea* Hochst.ex.A Rich  **Shrub** | Malvaceae | | Lenquata  (Ay43) | | | | Hu | | | Dandruff | **Bark F:** Washed the hair by the bark of *Grewia ferruginea* with water*.* | | Dermal |
|  |  |  |  |  |  |  |  | Li | | | Expel placenta | **Bark F:** The insider part of the bark *Grewia ferruginea* is pilled and given to animals. | | Oral |
|  |  |  |  |  |  |  |  | Li | | | Rabies | **Root D:** The dried root of *Grewiaferruginea* with the root of *Rumex nervosus* and*Acanthus sennii* grounded together and mix with milk then given to dog. | | Oral |
| 37 | *Hibiscus macranthus* Hochst. Rich. **Shrub** | Malvaceae | | Nacha  (Ad47) | | | | Hu | | | Syphilis | **Root D:** The root is pounded and mixes with oil or Vaseline and applied on the affected part of the body. | | Dermal |
| 38 | *Jasminum grandiflorum*L. **Shrub** | Oleaceae | | Tenbelel  (Ad9) | | | | Hu | | | Jaundice | **Leaf F:** The fresh leaves are crushed, grounded with water then take one glass for a weak before food in the morning. | | Oral. |
|  |  |  |  |  |  |  |  | Hu | | | Rheumatism | **Leaf and root F:** Root and leaf of *Jasminum abyssinicum* will be mixed with leaves of lemon then boiled in water and drink. | | Oral |
|  |  |  |  |  |  |  |  | Hu | | | Wound (Liffee) | **Leaf F:** fresh leaves and young bud are crushed, mix with butter then applied on the affected body part. | | Dermal |
|  |  | | *Table1: Continued* | | | | | | |  |  | |  |  |
| **No** | **Botanical name and plant habit** | **Family name** | | **Local name**  **and collection No** | | | | **Used for** | | | **Ailments** | **Part used and mode of preparation of the remedies** | | **Route of application** |
| 39 | *Juniperus procera* Hochst ex. Engl. **Tree** | Cuppressaceae | | Yeabeshatid  (Ay57) | | | | Hu | | | Wound | **Leaf F**: fresh leaves of *Juniperus procera* are sequenzed and applied on to the wound. | | Dermal |
| 40 | *Justicia schimperiana* (Hochst. ex Nees)  T.Anders. **Shrup** | Acanthaceae | | Smiza  (Ge64) | | | | HL | | | Rabies | **Root and Leaf D**: Root and leaf of *Justica schimperiana* will be pounded together and mixed with water and drunk. | | Oral |
|  |  |  |  |  |  |  |  | Hu | | | Gonorrhea | **Root F:** Root of *Justica schimperiana* together with leaf of *Lepidium sativum* pounded and drunk the solution with water. | | Oral |
|  |  |  |  |  |  |  |  | Hu | | | Wound  (Gormit) | **Leaf F:** fresh leaves of *Justicia schimperiana are* Crushed and pounded and mix with salt then creamed on wound until recovery. | | Dermal |
|  |  |  |  |  |  |  |  | HL | | | Diarrhea | **Leaf F:** Leaves of *Justicia schimperiana* smashed with water and the juice is consumed by human and animal in the morning before food. | | Oral |
| 41 | *Kalanchoe laciniata* L. **Herb** | Crassulaceae | | Andawula  (Ay27) | | | | Hu | | | Nasal bleeding | **Root and Leaf D:** Root and leaf of *Kalanchoe Petitiana* are powdered and sniffed. | | Nasal |
|  |  |  | |  | | | |  | | |  |  | |  |
|  |  |  | | *Table1: Continued* | | | | | | |  |  | |  |
| **No** | **Botanical name and plant habit** | **Family name** | | **Local name**  **and collection No** | | | | **Used for** | | | **Ailments** | **Part used and mode of preparation of the remedies** | | **Route of application** |
|  |  |  | |  | | | | Li | | | Body swelling(limsha) | **Steam F:** The steams are heated by fire and as soon as applied on swell part of cattle. | | Dermal |
|  |  |  |  |  |  |  |  | Hu | | | Leg swelling | **Steam F:** Fresh Steam *Kalanchoe laciniata* is inserted in to the fire and the hot steam put on the affected part. | | Dermal |
| 42 | *Lepidium sativum* L*.***Herb** | Brassicaceae | | Feto (Ge38) | | | | Hu | | | Bellyache | **Seed D:** seed of *Lepidium sativum* is grounded, mix with honey and drink by water. | | Oral |
|  |  |  | |  | | | | Hu | | | Malaria | **Seed D:** The seed of *Lepidium sativum* with bulb *Allium sativum* is pounded together and mixed with little water and drunk with water or eaten by Injera. | | Oral |
|  |  |  |  |  |  |  |  | Hu | | | Wound | **Seed D:** the seeds are grounded and mix with water then tied on the wound. | | Dermal |
|  |  |  |  |  |  |  |  | HL | | | Cough | **Seed D:** Dried seed crushed and mix with honey then drink by water for human and given with water for cattle. | | Oral |
| 43 | *Linum usitatissimum.* L **Herb** | Linaceae | | Telba  (Ge49) | | | | Hu | | | Hibiscus | Seed D: Dried seed of *Linum usitatissimum* is inserted in to the eye when dust particle inter in to the eye. | | Eye |
|  |  |  | | *Table1: Continued* | | | | | | |  |  | |  |
| **No** | **Botanical name and plant habit** | **Family name** | | **Local name**  **and collection No** | | | | **Used for** | | | **Ailments** | **Part used and mode of preparation of the remedies** | | **Route of application** |
|  |  |  | |  | | | | Hu | | | Gastritis | **Seed D:** Dried seeds are grounded, boiled then it will be eaten with Injera as *wot*. | | Oral |
| 44 | *Lippia abisinica* Koch*.***Shrub** | Verbenaceae | | Kesiy  (Ay29) | | | | Hu | | | Impotency for men | **Root D:**Grinding the root and mix with butter, then applying on the penis. | | Dermal |
|  |  |  |  |  |  |  |  | Hu | | | Fibril illness | **Leaf F:** The freshly leaf and immature stem of the plant is pounded and mixed with small amount of coffee and then drunk. | | Oral |
|  |  |  |  |  |  |  |  | Hu | | | Headache | **Leaf F:** Fresh leaves of *Lippia adoensis* is  sequenced with water and drunk. | | Oral |
| 45 | *Lupines albus .L*  **Herb** | Fabaceae | | Gibto  (Ad77) | | | | Hu | | | Hypertension | **Seed D:** The dried seed of *Lupines albus* is roasted and one spoon of the flour mixed with one glass of water then drunk during pain. | | Oral |
| 46 | *Solanumlycopersicum* L.**Herb** | Solanaceae | | Timatim  (Ay25) | | | | Hu | | | Hemorrhoid | **Fruit F:** the fruit is crushed into piece and held or tied on the pain area. | | Anal |
|  |  |  |  |  |  |  |  | Hu | | | Leech | **Leaf DF:** The leaf of *Lycopersicones cul antum* is ground with salt and added though nose. | | Nasal |
|  | | | | | | |  | | | |  |  |  |  |
|  | | | | | | |  |  |  |  |  |  |  |  |
|  |  |  | | *Table1: Continued* | | | | | | |  |  | |  |
| **No** | **Botanical name and plant habit** | **Family name** | | **Local name**  **and collection No** | | | | **Used for** | | | **Ailments** | **Part used and mode of preparation of the remedies** | | **Route of application** |
| 47 | *Malva verticillata* L.**Herb** | Malvaceae | | Tult  (Ad78) | | | | Hu | | | Diarrhea | **Root F:** Fresh root of *Malva verticillata* is ground with the seed of *Lepidium sativum* then drunk the solution with water. | | Oral |
| 48 | *Maytenussenegalensis* Rich. Wilczek. **Shrup** | Celastraceae | | Atat (Ad73) | | | | Hu | | | Kidney problem | **Root F/D:** The root of *Maytenussene galensis* with *Croton macrostachyus* are crushed mixed with milk and one cup drunk every morning before food until recovery. | | Oral |
| 49 | *Mimusops kummel* Benth . **Tree** | Sapotaceae | | Eshe  (Ad72) | | | | Hu | | | Diarrhea | **Fruit F:** Freshly fruit of *Mimusops kummel* will be eaten. | | Oral |
| 50 | *Myrsine africana*  L.**Shrup** | Primulaceae | | Kechemo  (Ge36) | | | | Hu | | | Tapeworm | **Fruit D:** Dried fruit is pound and mix with water then drink in the morning before food after that stay for six to seven hour without food. | | Oral |
| 51 | *Myrica salicifolia* Hochst. ex A. Rich **Tree**. | Myrsinaceae | | Shinet  (Ge51) | | | | Hu | | | Tonsillitis | **Bark D:** Dried bark will be crushed, mix with water and drunk. | | Oral |
|  |  |  |  |  |  |  |  | Li | | | Epilepsy (kumegna) | **Bark D:** Dried Bark of *Myrica salicifolia* is pounded and mix with water and given to  cattle through nose. | | nasal |
|  | | | | | | | |  | | | | | | |
|  |  |  | | *Table1: Continued* | | | | | | |  |  | |  |
| **No** | **Botanical name and plant habit** | **Family name** | | **Local name**  **and No of collaction** | | | | **Used for** | | | **Ailments** | **Part used and mode of preparation of the remedies** | | **Route of application** |
| 52 | *Ocimum gratissimum* Koth. **Shrup** | Lamiaceae | | Damakesy  (Ad70) | | | | Hu | | | Febrile illness | **Leaf D:** dried leaves of *Ocimum urticifolium* are powdered with coffee and drink before food in the morning. | | Oral |
| 53 | *Olea europaea L.*  **Tree** | Oleaceae | | Woira  (Ad67) | | | | Hu | | | Hemorrhoid | **Bark F:** The bark of *Olea europaea* will be heated on fire and held on the pain area. | | Dermal |
|  |  |  |  |  |  |  |  | Hu | | | Epilepsy | **Leaf D:** the dried leaves apply on the fire and the smoke will be inhaled. | | Nasal |
| 54 | *Osyris quadripartita* Benth. **Shrub** | Santalaceae | | | Keret  (Ge65) | | | Hu | | | Wound  (Lifee) | **Leaf and root FD:** the leaves and root are crushed roasted together then mix with butter after that apply on the affected body part. | | Dermal |
| 55 | *Otostegia integrifolia* Benth.  **Shrub** | Lamiaceae | | | Tunjut  (Ge59) | | | Hu | | | Nasal bleeding  (nesr) | **Leaf F:** Fresh leaves are squeezed and inhaled through nasal opening. | | Nasal |
|  |  |  |  |  |  |  |  | Hu | | | Stomachache | **Steam and leaf F:** Steam and leaf of *Otostegia integrifolia* are given for chewing and swallow the solution. | | Oral |
|  |  |  |  |  |  |  |  | Hu | | | Diarrhea | **Root F:** Root of *Otostegia integrifolia* is chewed with salt and swallowed the solution. | | Oral |
|  |  |  | | |  | | |  | | |  |  | |  |
| *Table1: Continued* | | | | | |  |  | |  |  |  |  |  |  |
| **No** | **Botanical name and plant habit** | **Family name** | | | **Local name**  **and collectionNo** | | | **Used for** | | | **Ailments** | **Part used and mode of preparation of the remedies** | | **Route of application** |
| 56 | *Phoenix reclinata* Jacq. **Shrub** | Arecaceae | | | Seniel  (Ay20) | | | HL | | | Intestinal parasite | **Root F:** The fresh root juice of *Phoenix reclinata* is mixed with water and given tohuman and livestock. | | Oral |
| 57 | *Persea americana* Mill. **Tree** | Lauraceae | | | Avocado  (Ay15) | | | Hu | | | Kidney infection | **Leaf FD:** The leaves of *Persea Americana* are boiled and the liquid portion is drunk until recovery. | | Oral |
| 58 | *Phytolacca dodecandra*  L’Herit. **Climber** | Phytolaccaceae | | | Endod  (Ay19) | | | Hu | | | Gonorrhea | **Root FD*:***Roots of *Phytolacca dodecandra* and *Croton macrostachyus* are powdered together and drunk with coffee. | | Oral |
|  |  |  |  |  |  |  |  | HL | | | Rabies | **Root D:** Dried root of *Phytolacca dodecandra* will be grounded and the powder drunk with locally made alcohol (*tella*) for human or the leaves are Crushed, mixed withmilk then given to dog in order to avoid contamination. | | Oral |
|  |  |  |  |  |  |  |  | Hu | | | Anthrax | **Leaf FD:** The leaves of *Phytolacca dodecandra* is crushed and mixed with water then drunk | | Oral |
| 59 | *Plantago lanceolata* L. Herb | Plantaginaceae | | | Worteb  (Ay33) | | | Hu | | | Wound and  Bleeding | **Leaf F:** The leaves of *Plantago lanceolata* are crushed and the powder tied or mix with water wash until recovery. | | Dermal |
|  |  |  | | | *Table1: Continued* | | | | | |  |  | |  |
| **No** | **Botanical name and plant habit** | **Family name** | | | **Local name**  **and collectionNo** | | | **Used for** | | | **Ailments** | **Part used and mode of preparation of the remedies** | | **Route of application** |
|  |  |  | | |  | | | Hu | | | Coccidosis | **Leaf FD:** The leaves of the *Plantago lanceolata* are crushed and the powder mix with water then drunk. | | Oral |
| 60 | *Pterolobium stellatum* Forsk. Brenan. **Climber** | Fabaceae | | | Kentafa  (Ay53) | | | Hu | | | Epilepsy | **Root FD:** Root of *Pterolobium stellatum* and root of *Ruta chalepensis* are powdered together and sniffed. | | Nasal |
|  |  |  |  |  |  |  |  | Hu | | | Jaundice | **Root D:** Root of *Pterolobium stellatum* isdried, powdered and one spoon of the powder is mixed with half cup of local alcohol and consumed it*.* | | Oral |
| 61 | *Rhamnus prinoides* L**Shrub** | Rhamnaceae | | | Gesho  (Ay22) | | | Hu | | | Snake bite | **Root F:** The fresh root of *Rhamnus prinoides is* chewed during pain time. | | Oral |
|  |  |  |  |  |  |  |  |  |  |  | Wound  (kufign) | **Seed FD:** The seeds of *Rhamnus prinoides* are grounded and applied to the affected area. | | Dermal |
| 62 | *Rhus retinorrhoae* Krauss. **Tree** | Ancardaceae | | | Telem  (Ge45) | | | Hu | | | Jaundice | **Leaf D:** The leaves of *Rhus retinorrhoae* are crushed, grounded and boiled with water then drunk with local alcohol (*arekie*) after cooled. | | Oral |
| 63 | *Ricinus communis* L. **Shrub** | Euphorbiaceae | | | Chakima/ Gulo  (Ay32) | | | Hu | | | Scabies | **Seed D:** Seed of *Ricinus communis* is crushed and mix with butter then creamed on the skin. | | Dermal |
|  | | | | | *Table1: Continued* | | | | | | | | | |
| **No** | **Botanical name and plant habit** | **Family name** | | | **Local name**  **and collectionNo** | | | **Used for** | | | **Ailments** | **Part used and mode of preparation of the remedies** | | **Route of application** |
|  |  |  | | |  | | |  | | | Hemorrhoid | **Leaf F:** The leaves will be crushed, chopped and mix with butter after that applies on the affected part. | | Dermal |
|  |  |  | | |  | | | Hu | | | Stomachache | **Fruit F:** The fresh fruit of *Rosa abyssinica* are chewing and swallowed the decoction. | | Oral |
| 65 | *Rumex abyssinicus* Jacq. **Herb** | Polygonaceae | | | Mekimeko  (Ge6) | | | Hu | | | *yewurch* | **Root D:** Root of *Rumex abyssinica* and *Trichodesma zeylanicum* arecrushed and ground together. Then stay the powder in one litter of local alcohol (*Arekie*) for seven days. After that half of cup or one *melekiya* will be drunk until it will be finished before food. | | Oral |
| 66 | *Rumex nervosus* Vahl. **Shrub** | Polygonaceae | | | Enbuacho  (Ay11) | | | HL | | | Rabies | **Root D:** The root of *Rumex nervosus* and *Phytolacca dodecandra* are pounded together then mix with milk and consumed it. | | Oral |
|  |  |  |  |  |  |  |  | Hu | | | Snake bite | **Leaf F:** The leaf of *Rumex nervosus* is chewing and swallowing the solution during time of bite. | | Oral |
|  |  |  |  |  |  |  |  | Hu | | | Itching | **Leaf F:** The leaf of *Rumex nervosus* is, crushed and pounded then creamed on wounded part with salt. | | Dermal |
|  |  |  | | | *Table1: Continued* | | | | | |  |  | |  |
| **No** | **Botanical name and plant habit** | **Family name** | | | **Local name**  **and collectionNo** | | | **Used for** | | | **Ailments** | **Part used and mode of preparation of the remedies** | | **Route of application** |
| 67 | *Ruta chalepensis* L.**Herb** | Rutaceae | | | Tenadam  (Ay31) | | | Hu | | | Stomachache | **Leaf F:** The leaf of *Ruta chalepensis* Squeezed and drunk the juice during ach. | | Oral |
|  |  |  | | |  | | | Hu | | | Epilepsy | **Seed and leaf F:** The seed and leaf of *Ruta chalepensis* with *Allium sativum* mix and pounded together then sniffed at the sickness time. | | Nasal |
|  |  |  |  |  |  |  |  | Hu | | | Headache | **Leaf F:** The leaf of *Ruta chalepensis* Squeezed and drunk with coffee. | | Oral |
| 68 | *Schefifera abyssinica* Hochst. ex A. Rich.**Tree** | Araliaceae | | | Getem  (Ay18) | | | Li | | | External parasites | **Leaf F:** Fresh leaves of *Schefifera abyssinica* are pounded and the powder mixed with butter and creamed on affected skin. | | Dermal |
| 69 | *Sida tenuicarpa* Vollesen. **Shrub** | Malvaceae | | | Chifirg  (Ge60) | | | Hu | | | Bone tumors | **Root and leaf FD:** The leaf and root of *Sida schimperi* is pounded and the powder then applied on the affected part. | | Dermal |
| 70 | *Solanum marginatum* L.**Herb** | Solanaceae | | | Gebre enbuay  (Ay13) | | | Hu | | | Tonsillitis | **Fruit F:** The Fruit liquid of *Solanum marginatum* and bulb of *Allium sativum* are pounded together and given to human with honey for 2-3 days. | | Oral |
|  | | | | | *Table1: Continued* | | | | | | | | | |
| **No** | **Botanical name and plant habit** | **Family name** | | | **Local name**  **and collectionNo** | | | **Used for** | | | **Ailments** | **Part used and mode of preparation of the remedies** | | **Route of application** |
|  |  |  | | |  | | | Hu | | | Abortion | **Root F:** The *Solanum marginatum* seed and root of *Arundo donax* crushed and grounded together then mixed by water and drunk it with a cup of tea for seven days. | | Oral |
| 71 | *Solanum incanum* L.**Herb** | Solanaceae | | | Enbuay  (Ay68) | | | Hu | | | Itching | **Fruit F:** The fruit juice of *Solanum incanum* is squeezed between palms and the latex is applied on the wound. After five to seven hour the wound is washed with water until recovery. | | Dermal |
| 72 | *Syzygium guineense* Willd. **T** | Myrtaceae | | | Dokima  (Ay56) | | | Li | | | Bloating | **Leaf F:** The leaves *Syzygium guineense* is crushed, pounded and given with water. | | Oral |
| 73 | *Terminalia schimperiana* Willd. **Tree** | Combretaceae | | | Abalo  (Ay66) | | | Hu | | | Eczema | **Root and Leaf F:** The fresh Root and leaves of *Terminalia schimperiana* are pounded and mixed with butter and applied on the wound. | | Dermal |
| 74 | *Trichodesma zeylanicum.*  **Herb** | Boraginaceae | | | Yewusha milas/Ay3 | | | Hu | | | *yewurch* | **Root D:** Root of *Trichodesma zeylanicum* and *Trichodesma zeylanicum* are crushed and ground together. Then stay the powder in one litter of local alcohol (*Arekie*). After that half of cup or one *melekiya* will be drunk until it will be finished before food. | | Oral |
| *Table1: Continued* | | | | | | | | |  |  |  |  |  |  |
| **No** | **Botanical name and plant habit** | **Family name** | | | | **Local name**  **and collection No** | | | **Used for** | | **Ailments** | **Part used and mode of preparation of the remedies** | | **Route of application** |
| 75 | *Urera hypselodendron* (Hochst.) ex A. Rich. **Climber** | Urticaceae | | | | Lankuso  (Ay23) | | | Hu | | Epilepsy | **Root D:** The dried root of *Urera hypselodendron* is grounded *and* one spoon of the powder is mixed with half cup of local alcohol and given to human*.* | | Oral |
| 76 | *Verbena officinalis* L. **Herb** | Verbenaceae | | | | Atuch  (Ay58) | | | Hu | | Stomachache, diarrhea | **Root F:** Root of *Verbena officinalis* is Chewed and swallowed the solution. | | Oral |
| 77 | *Vernonia amygdalina* Del. **Tree** | Astraceae | | | | Girawa  (Ay4) | | | HL | | Anthrax | **Leaf F:** Leaf of *Vernonia amygdalina* together with bulb of *Allium sativum* grounded and cooked then eaten with Injera before food. | | Oral |
|  |  |  |  |  |  |  |  |  | Li | | Eye disease | **Root F:** The root of *Vernonia amygdalina* are pounded and the liquid part added into the eye. | | Through the eye. |
| 78 | *Ximenia americana* L. **Shrub** | Olacaceae | | | | Enkoy  (Ay44) | | | Li | | Granule | **Stem bark FD:** The bark of *Ximenia Americana* is crushed, ground and creamed on the affected part. | | Dermal |
| 79 | *Zehneria scabra* (Linn. f.) **Cliber** | Cucurbitaceae | | | | Hareg resa  (Ad76) | | | Hu | | Fibril illness | **Above ground part F:** Above ground part of *Zehneria scabra* is boiled with water and fumigated the vapor during bed time. | | Nasal/ Oral |
|  |  |  | | | | *Table1: Continued* | | | | |  |  | |  |
| **No** | **Botanical name and plant habit** | **Family name** | | | | **Local name**  **and collection No** | | | **Used for** | | **Ailments** | **Part used and mode of preparation of the remedies** | | **Route of application** |
|  |  |  | | | |  | | | HL | | Skin rash | **Above ground part F:** Above ground part is immersed in hot water and rubbed to the affected skin of human animals. | | Dermal |
| 80 | *Zingiber oﬃcinale* Rosc. Herb | Zingiberaceae | | | | Zingibl  (Ay35) | | | Hu | | Common cold | **Tuber FD:** The tuber of *Zingiber oﬃcinale* is crushed boiled with coffee and drunk. | | Oral |

**Table 2:** **Habitat of Medicinal Plant Species in the Study Area**

| Species | Habitat | Species | Habitat | Species | Habitat |
| --- | --- | --- | --- | --- | --- |
| *Acacia abyssinica* | Forest | *Acanthus sennii* | Forest | *Achyranthes aspera* | Forest |
| *Acokanthera schimperi* | Forest | *Allium sativium* | Home garden | *Aloe macrocarpa* | Forest |
| *Arundo donax* | Home garden | *Argemone Mexicana* | Grazing land | *Asparagus africanus* | Forest |
| *Bersama abyssinica* | Forest | *Buddleja polystachya* | Forest | *Capsicum annuum* | Agricultural field |
| *Carissa spinarum* | Forest | *Catha edulis* | Home garden | *Citrus aurantifolia* | Home garden |
| *Clausena anisata* | Forest | *Clematis simensis* | Forest | *Clerodendrum Myricoides* | Forest |
| *Coffea arabica* | Home garden | *Cordia africana* | Agricultural field | *Croton macrostachyus* | Agricultural field |
| *Cucumis ficifolius* | Forest | *Cucurbita pepo* | Home garden | *Datura stramonium* | Agricultural field |
| *Dodonaea angustifolia* | Forest | *Dombeya torrid* | Forest | *Dovyalis abyssinica* | Forest |
| *Embelia schimperi* | Forest | *Eucalyptus globules* | Agricultural & Road side | *Euclea racemosa* | Forest |
| *Euphorbia abyssinica* | Forest | *Euphorbia tirucallia* | Road side | *Ficus vasta* | Forest |
| *Foeniculum vulgare* | Around home | *Grewia ferruginea* | Forest | *Hibiscus macranthus* | Forest |
| *Jasminum abyssinicum* | Forest | *Juniperus procera* | Forest | *Justicia schimperiana* | Road side |
| *Kalanchoe laciniata* | Grazing land | *Lepidium sativum* | Agricultural field | *Linum usitatissimum* | Agricultural field |
| *Lippia adoensis* | Forest | *Lupines albus* | Agricultural field | *Lycopersicumesculantum* | Home garden |
| *Malva verticillata* | Grazing land | *Maytenussenegalensis* | Forest | *Mimusops kummel* | River side |
| *Myrsine Africana* | Forest | *Myrica salicifolia* | Forest | *Ocimum urticifolium* | Forest |
| *Olea europaea* | Agricultural field | *Osyris quadripartita* | Forest | *Otostegia integrifolia* | Forest |
| *Phoenix reclinata* | River side | *Persea Americana* | Home garden | *Phytolacca dodecandra* | Forest |

*Table 2: Continued*

| **Species** | **Habitat** | **Species** | **Habitat** | **Species** | **Habitat** |
| --- | --- | --- | --- | --- | --- |
| *Plantago lanceolata* | Agricult ural field | *Pterolobium stellatum* | Forest | *Rhamnus prinoides* | Home garden |
| *Rhus retinorrhoae* | Forest | *Ricinus communis* | Agricult ural field | *Rosa abyssinica* | Forest |
| *Rumex abyssinicus* | Forest | *Rumex nepalensis* | Grazing land | *Rumex nervosus* | Forest |
| *Ruta chalepensis* | Home garden | *Schefifera abyssinica* | Forest | *Sida tenuicarpa* | Grazing land |
| *Solanum marginatum* | Forest | *Solanum incanum* | Forest | *Syzygium guineense* | River side |
| *TerminaliaSchimp eriana* | Forest | *Trichodesma zeylanicum* | Agricult ural field | *Urera hypselodendron* | Forest |
| *Verbena officinalis* | Grazing land | *Vernonia amygdalina* | Agricult ural field | *Ximenia americana* | Forest |
| *Zehneria scabra* | Home garden | *Zingiber o cinale* | Home garden | *Euphorbia abyssinica* | Around home |

**Table 3: Health Problems Treated by Traditional Medicinal Plants in the Study Area**

| **English name** | **Local Name** | **English name** | **Local Name** |
| --- | --- | --- | --- |
| Stomachache | Yehodkurtet | Common cold | Gunfan |
| Insect bite | Yebab niksha | Impotency | Ywsibdikmet |
| Rabies | Yewsha beshita | Abortion | Shotelay |
| Wound | Liffe | Gonorrhea | Chebt |
| Bleeding | Yesewnet Medmat | Toothache | Tirs kurtimat |
| Liver Problem | Gubet | Ascariasis | Wosfat |
| Malaria | Weba | Tonsillitis | Tonsil |
| Hibiscus | Kontir | Diarrhea | Tekimat |
| Hemorrhoid | Kintarot | Fire burn | Yesat makatel |
| Wound | Kusil | Herpes | Almaze balchera |
| Asthma | Asm | Febrile Illness | Mich |
| Athletes foot | Yegir meshitet | Expel placenta | Yengdelj sizegey |
| Skin rash | **Chifee** | Tapeworm | Kosso |
| Granule | Bigr | Dandruff | Forefor |
| Wart | Chirt | Eczema | Chiffe |
| Abdominal pain | Yehodhimem | Syphilis | Kitegn |
| Intestinal parasites | Yeanjet beshita | Rheumatism | Kurtmat |
| Jaundice | Yewofitu | Wound | Gormit |
| Wound | Kunchir | Nasal bleeding | Nesir |
| Cough | Sal | Body swelling | Limsha |
| Urinary Retention | Shintemat | Leg swelling | Yegir ebtet |
| Gastritis | Chenguara | Cold disease | Yewurch |
| Hypertension | Yedemgifit | Itching | Ekek |
| Leech | Alikt | Bone tumors | Nekersa |
| Kidney problem | Yekulali himet | Bloating | Yehode menifat |
| Anthrax | Kurba | Ache | Kufign |
| Epilepsy | Yemitl himem | Scabies | Kerkir |

**Table 4: Total Number of Informants in the Study Area in Each *Kebele***

| **Name of Kebele** | **Traditional Healers** | | | **Nontraditional Healers** | | | **Total Informants** | | |
| --- | --- | --- | --- | --- | --- | --- | --- | --- | --- |
|  | Male | Female | Total | Male | Female | Total | Male | Female | Total |
| Ayenbirhan | 11 | 5 | 16 | 9 | 7 | 16 | 20 | 12 | 32 |
| Gedamabo | 13 | 4 | 17 | 14 | 4 | 18 | 27 | 8 | 35 |
| Addisalem | 10 | 2 | 12 | 13 | 8 | 21 | 23 | 10 | 33 |
| Total | 34 | 11 | 45 | 36 | 19 | 55 | 70 | 30 | 100 |
